# Variation in the Fatty Acid and Amino Acid Profiles of Pasteurized Liquid Whole Hen Egg Products Stored in Four Types of Packaging

**DOI:** 10.3390/ani12212990

**Published:** 2022-10-30

**Authors:** Vjaceslavs Kocetkovs, Vitalijs Radenkovs, Karina Juhnevica-Radenkova, Sandra Muizniece-Brasava

**Affiliations:** 1 Faculty of Food Technology, Latvia University of Life Sciences and Technologies, LV-3004 Jelgava, Latvia; 2 Processing and Biochemistry Department, Institute of Horticulture, LV-3701 Dobele, Latvia; 3 Division of Agronomic Analysis, Research Laboratory of Biotechnology, Latvia University of Life Sciences and Technologies, LV-3002 Jelgava, Latvia

**Keywords:** liquid egg product, bio-based packaging, essential fatty acids, plastic, Tetra Rex, high-density polyethylene

## Abstract

**Simple Summary:**

Getting the packaging right in a competitive landscape such as the food industry is essential. Packaging along with its ability to preserve the nutritional composition of products during shelf-life must also be robust, tamper-proof, and leak-free. Various types of packaging are currently used for marketing purposes, which play a role in safety and convenience, efficiency, and consumer information. The preservation of liquid whole egg products (LWEPs) quality is ensured by the application of materials such as high-density polyethylene (HDPE), polyethylene terephthalate (PET), and Tetra Rex (TR). To date, however, due to limitations in scientific evidence concerning the quality analysis and deterioration mechanism of LWEP during refrigerated storage, no unambiguous conclusions regarding the benefits of one packaging over another are possible to draw. The acquired results revealed the remarkable advantage of PET over other packaging materials in the ability to maintain polyunsaturated fatty acids and amino acid levels during 35 days of LWEP storage. In turn, the moisture loss induced by exfoliation of the internal polymer layers presented in TR and the hydrophilic nature of the ethylene vinyl alcohol layer in Doypack (stand-up pouches) was the main factor that caused substantial fluctuations in the level of fatty acids and amino acids during storage.

**Abstract:**

This study aimed to determine the ability of high-density polyethylene, polyethylene terephthalate, Tetra Rex^®^ Bio-based packaging, and Doypack (stand-up pouches) packaging to maintain the nutritional quality and safety of liquid whole egg products for 35 days of refrigerated storage. High-grade hen eggs were used for the preparation of liquid whole egg products (LWEPs). The conformity of eggs quality to grade A was supported by the initial screening of the raw materials’ physical–chemical attributes, which remained unchanged during the 25 days of storage. The obtained results indicated that the content of fatty acids in LWEPs was affected by both storage time and packaging material. However, the better preservation of monounsaturated fatty acids was achieved by polyethylene terephthalate, followed by high-density polyethylene packaging. Meanwhile, a statistically significant advantage of polyethylene terephthalate over other packaging materials was also confirmed regarding the maintenance of polyunsaturated fatty acids during 35 days of LWEPs storage. Relative fluctuations in the number of fatty acids in Tetra Rex^®^ Bio-based and Doypack-stored LWEPs revealed their disadvantages manifested by exfoliation of composite layers, which perhaps was the main cause of extensive moisture loss. Overall, due to superior barrier properties, polyethylene terephthalate packaging demonstrated better preservation of amino acids. Only as much as a 2.1% decrease was observed between the initial value and the 35th day of LWEP storage. From a microbiological standpoint, all materials demonstrated the ability to ensure the microbiological safety of products during 35 days of storage, as the maximum allowed limit of 10^5^ CFU g^−1^ was not exceeded.

## 1. Introduction

Hen’s eggs are considered one of nature’s most complete commodities because of their high nutritional value. Eggs are composed of various nutrients, vitamins, minerals, fatty acids (FAs), bioactive compounds and protein, making them one of the essential foods in human nutrition [1,2,3,4,5]. Furthermore, they are cheap and widely distributed in most countries, allowing people with low incomes to consume highly nutritional foods [6]. 

The shelf-life of eggs concerning physical–chemical quality attributes is affected by extrinsic factors such as environmental, production, and storage conditions along with intrinsic nutritional composition: mainly proteins, pH, and water activity [7]. Due to elevated moisture content and water activity, eggs and egg-derived products are characterized as highly perishable commodities [8]. Furthermore, the availability of FAs, especially monounsaturated (MUFA) and polyunsaturated (PUFA) FAs makes this product highly susceptible to oxidation. The quality rapidly deteriorates during the period from cracking to consumption [9]. To maintain the quality of egg-derived products during the entire storage period, proper packaging must be selected, and the eggs must be safely handled until processed. 

Currently, various types of packaging are used for marketing purposes, which play a role not only in safety but also in convenience, efficiency, and consumer information. The preservation of liquid whole egg products (LWEPs) quality is ensured by the application of materials such as high-density polyethylene (HDPE), polyethylene terephthalate (PET), and Tetra Rex (TR). However, scientific evidence to date is not enough to make univocal statements regarding the benefits of one packaging or another, as there are no reports published thus far regarding the quality analysis and deterioration mechanism of LWEP during refrigerated storage.

In addition to high nutritional value, eggs and their derived products must meet safety standards outlined by the Commission Regulation (EC) No 589/2008 and No 2073/2005 [10,11]. Therefore, technologies presently utilized, i.e., pasteurization, cooling, freezing, etc., are generally aimed at ensuring the safety and extension of the shelf-life of the stored products. Pasteurization is largely utilized within various subsectors of the food industry and is found to be effective in the inactivation of foodborne pathogens, toxins, and other detrimental constituents; this currently allows for considerably extended storage time of the products risking food poisoning [12,13]. However, along with its advantages, pasteurization substantially affects the reduction of valuable nutrients and loss of functionality due to its elevated temperature (usually ≥82 °C). High-temperature food treatment can impair nutritional quality, appearance, flavor, etc., [14,15,16]. Furthermore, this type of processing is not always valid for certain products, especially those containing compounds highly sensitive to environmental conditions, such as proteins.

According to the guidelines prepared by the Food Safety and Inspection Service [17], a compromise between quality and safety can be achieved using mild and time-controlled thermal processing. Pasteurization temperature at which the most viable cells of *Salmonella* spp. become inactivated should correspond to a temperature of 61.6 to 65.0 °C with a minimum of 3.5 min of exposure time. This statement was reinforced by an observation made by Lopes et al. [18]. Following these guidelines, along with properly selecting packaging material, is likely to result in sufficient shelf-life duration of LWEPs under reasonable operational costs without affecting nutritional quality and functionality.

The objective of the present study was to elucidate the influence of storage time and the type of packaging, i.e., high-density polyethylene (HDPE), polyethylene terephthalate (PET), Tetra Rex^®^ Bio-based (TR), and Doypack with ethylene vinyl alcohol (EVOH) layer (DP), on the dynamics change in the content of FAs, AAs and total microorganism count (TPC) in liquid whole egg products during 35 days of refrigerated storage.

## 2. Materials and Methods

### 2.1. Initial Screening of Physical Attributes of Hen Eggs

Fresh eggs of Lohmann Brown laying hens (29 weeks old) (Grade A, large size, the same diet and housing) not longer than 5 days after laying were collected from JSC “Balticovo” farm. For subsequent processing, the eggs were maintained for 1, 3, 5, and 30 days at 19 ± 1 °C temperature with 50% relative air humidity. At sampling, eggs were weighed and broken onto a flat surface where the height of the inner thick albumen and Haugh unit [19] was measured using an electronic albumen height digital Haugh tester ORKA (Bountiful, UT, United States). Measurement of the air cell size was performed using an ovoscope (Ovolux, Masalles, Spain). The pH of eggs was determined using a pH-meter Jenway 3510 Benchtop pH Meter (Barloworld Scientific, Staffordshire, United Kingdom) according to ISO 1842:1991. The eggshell thickness was determined using a disc micrometer-mechanical counter Mitutoyo 223–101 (Kawasaki, Japan) equipped with an aluminum compression disc 7.62 cm in diameter. Dry matter was analyzed using a Shimadzu MOC-120H (Shimadzu Corporation, Tokyo, Japan) moisture analyzer. 

### 2.2. Preparation of Liquid Whole Egg Products for Subsequent Storage

**Figure 1:**
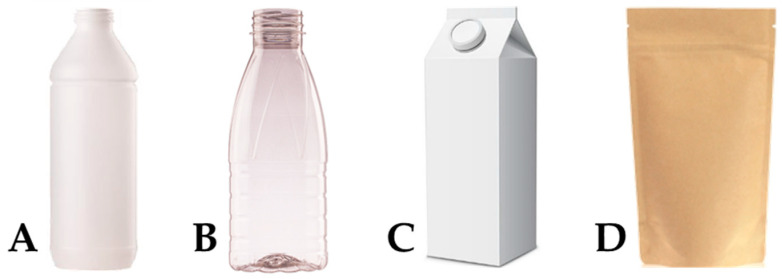
Packaging materials used for liquid whole egg product storage. (**A**)—HDPE packaging made of high-density polyethylene. (**B**)—PET packaging made of polyethylene terephthalate. (**C**)—Tetra Rex^®^ Bio-based multi-layer packaging made of carton and low-density polyethylene (LDPE), and HDPE derived from sugar cane polymers. (**D**)—Doypack multi-layer packaging made of carton and vinyl alcohol-co-ethylene (EVOH) layer.

Approximately 300 eggs per breed were harvested for the preparation of LWEPs. Liquid egg mass was pasteurized using the plate pasteurizer Ovobel AR56SH (Brugge, Belgium). The pasteurization process lasted for 6 min at 68 ± 1 °C temperature. Following pasteurization, the LWEPs were packed into four types of packaging with a volume of 0.5 L, i.e., high-density polyethylene (HDPE), polyethylene terephthalate (PET) bottles, Tetra Rex^®^ Bio-based packs (TR), and multi-layer Doypack EVOH bags (DP) (Figure 1). The LWEPs were maintained at 4 ± 1 °C for 35 days. 

### 2.3. Chemicals and Reagents

A mixture of C_4_-C_24_ FA methyl esters (FAMEs) with purity ≥99.0% and cholesterol (C_27_H_46_O) of a purity ≥99.0% were acquired from Sigma-Aldrich Chemie Ltd., (St. Louis, MO, USA). Sodium hydroxide (NaOH), potassium hydroxide (KOH), phenolphthalein (C_20_H_14_O_4_), and 0.5M trimethylphenylammonium hydroxide solution (CH_3_)_3_N(OH)C_6_H_5_ (TMPAH) in methanol (MeOH) for GC derivatization of reagent grade were purchased from Sigma-Aldrich Chemie Ltd. HPLC grade MeOH, *n*-hexane (C_6_H_14_) and pyridine (C_5_H_5_N) were purchased from Sigma-Aldrich Chemie Ltd. Diethyl ether ((C_2_H_5_)_2_O)) (purity, ≥99.5%) was obtained from Chempur (Piekary Śląskie, Silesia, Poland). Derivatization agent N,O-bis(trimethylsilyl)trifluoroacetamide (BSTFA) was obtained from Supelco, Bellefonte, PA, USA. The ultrapure water was produced using the reverse osmosis PureLab Flex Elga water purification system (Veolia Water Technologies, Paris, France).

### 2.4. Preparation of Liquid Whole Egg Lipid Fraction 

Preparation of lipid fraction was carried out following the procedures described by Radenkovs et al. [20] with minor modifications. For the release of bound forms of FAs, 10% (*w*/*v*) KOH dissolved in 80% MeOH (MeOH:H_2_O ratio 80:20 *v*/*v*) was used. In the supremacy of MeOH, this approach allows the process of hydrolysis and release of FAs to be performed more efficiently. Briefly, triplicate samples of 3 ± 0.1 g of egg were weighed in 50 mL reagent bottles with screw caps. For the hydrolysis, 30 mL of prepared methanolic KOH was added to each egg sample, and the mixture was subjected to incubation in a water bath “TW8” (Julabo^®^, Saalbach-Hinterglemm, Germany) at 65 °C temperature for 3 h. After hydrolysis, the release of FAs from the salt form was performed by shifting the pH of the medium from alkaline to acidic, by adding 3.5 mL HCl (6 M) or until the pH 2.0. The extraction of the lipophilic fraction was accomplished by liquid–liquid phase separation using *n*-hexane as a solvent. Hydrolysates cooled to ambient temperature (22 ± 1 °C) were quantitatively transferred to Falcon 50 mL conical centrifuge tubes (Sarstedt AG & Co. KG, Nümbrecht, Germany), and 10 mL of *n*-hexane was added to each tube, followed by vortex-mixing for 1 min. Separation of the layers was performed by centrifugation at 4500 rpm (3169× *g*) for 10 min in a “Sigma, 2-16KC” centrifuge (Osterode near Harz, Germany). The top *n*-hexane layer was separated and collected. The extraction procedure was repeated three times. The resulting lipophilic fraction (30 mL) was further evaporated using a “Laborota 4002” rotary evaporator (Heidolph, Swabia, Germany) at 65 °C, and the dry fraction was then redissolved in 5 mL of *n*-hexane and filtered through a PTFE membrane filter with a pore size of 0.45 µm. The filtrates were quantitatively transferred to 20 mL scintillation glass vials and then subjected to drying under a gentle stream of N_2_ to complete dryness. Dry residues were weighed to determine the yield of lipids and kept at a temperature of −18 ± 1 °C until further analysis and use, a maximum of five weeks. Before GC-MS analysis, obtained dry lipid fractions were reconstituted in 5 mL pyridine.

### 2.5. Preparation of Fatty Acids for GC-MS Analysis 

The TMPAH reagent was applied as a methylation agent of the functional groups to obtain volatile FAMEs derivatives. The methylation procedure was performed following the methodology described by the American Society for Testing and Materials [21]. 

### 2.6. The GC Conditions for FAMEs Analysis

**Figure 2:**
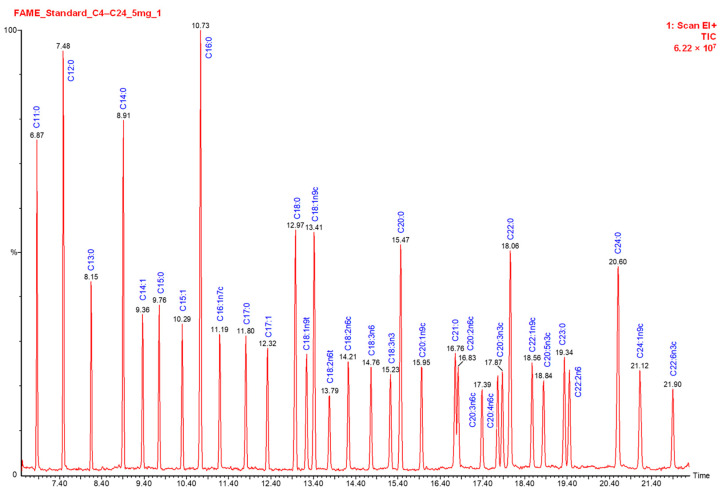
Chromatographic separation of C_4_-C_24_ FA methyl ester standards with reference to their retention times: 6.87 min (C11:0—undecanoic acid), 7.48 min (C12:0—dodecanoic acid), 8.15 min (C13:0—tridecanoic acid), 8.91 min (C14:0—tetradecanoic acid), 9.36 min (C14:1—tetradecenoic acid), 9.76 min (C15:0—pentadecanoic acid), 10.29 min (C15:1—pentadecenoic acid), 10.73 min (C16:0—hexadecanoic acid), 11.19 min (C16:1n7c—hexadecenoic acid), 11.80 min (C17:0—heptadecanoic acid), 12.32 min (C17:1—heptadecenoic acid), 12.97 min (C18:0—octadecanoic acid), 13.24 min (C18:1n9t—octadecenoic acid), 13.41 min (C18:1n9c—octadecenoic acid), 14.21 min (C18:2n6c—octadecadienoic acid), 14.76 min (C18:3n6c—octadecatrienoic acid), 15.23 min (C18:3n3c—octadecatrienoic acid), 15.47 min (C20:0—eicosanoic acid), 15.95 min (C20:1n9c—eicosenoic acid), 16.76 min (C21:0—heneicosanoic acid), 16.83 min (C20:2n6c—eicosadienoic acid), 17.39 min (C20:3n6c—eicosatrienoic acid), 17.76 min (C20:4n6c—arachidonic acid), 18.06 min (C22:0—docosanoic acid), 18.56 min (C22:1n9c—erucic acid), 18.84 min (C20:5n3c—eicosapentaenoic acid), 19.34 min (C23:0—tricosanoic acid), 19.46 min (C22:2n6c—docosadienoic acid), 20.60 min (C24:0—tetracosanoic acid), 21.12 min (C24:1n9c—tetracosenoic acid), 21.90 min (C22:6n3c—docosahexaenoic acid).

The analysis of FAMEs (Figure 2) was carried out on a “Clarus 600” system PerkinElmer, Inc. (Waltham, MA, USA) equipped with a quadrupole analyzer “Clarus 600 C” mass-selective detector (Waltham, MA, USA). The conditions were adopted from Radenkovs et al. [22]. 

### 2.7. The MS Conditions for FAMEs Detection

The MS conditions were set in accordance with the protocol provided by Radenkovs et al. [22]. 

### 2.8. Determination of Amino Acids

The preparation of LWEP samples for analysis of AAs was performed according to ISO 13910-2005. The analysis of AAs was conducted using Biochrom 30+ Automatic Amino Acid Analyzer (Biochrom, Cambridge, UK) in accordance with the protocol described by Summers et al. [23]. 

### 2.9. Microbiological Assessment 

Determination of the total microorganisms, mesophylic aerobic and facultative anaerobic microorganisms was performed according to ISO 4833:1:2012.

### 2.10. Statistical Analysis

The results obtained are shown as means ± standard deviation of the mean from three replicates (*n* = 3). A *p* value of <0.05 was used to denote significant differences between mean values determined using a one-way analysis of variance (ANOVA) and Duncan’s multiple range test performed using IBM^®^ SPSS^®^ Statistics version 20.0 (SPSS Inc., Chicago, IL, USA).

## 3. Results and Discussion

### 3.1. Changes in Physical Attributes of Non-Processed Eggs during Storage under Ambient Conditions

The purpose of the experiment was to identify changes in the physical–chemical attributes of non-processed eggs during 30 days of storage. Initial screening of the physical attributes of non-processed whole eggs revealed statistically significant (*p* ≤ 0.05) differences in selected criteria values (except for pH value) during 30 days of shelf-life at 19 ± 1 °C temperature and RH of 50% (Table 1). 

The importance of egg weight loss analysis as a direct and accurate metric to evaluate the quality and shelf-life of eggs has been highlighted by da Silva Pires et al. [24] Therefore, this quality trait has been taken critically to evaluate the dynamics of quality change during storage. It has been observed that the most substantial changes in physical attributes during storage were found to be egg weight. As seen, up to a 4.3% decrease in egg weight was marked after 10 days of storage compared with the initial value. The results are consistent with those of Khan et al. [25], indicating a fairly similar percentage reduction after 10 days of egg storage at 21 °C. Weight loss after 30 days of storage was found to be 10.1%, which is fairly higher than highlighted by Jones et al. [26] for unwashed eggs kept for 4 weeks under 22 °C temperature. Weight reduction is associated primarily with the loss of moisture along with CO_2_ due to the breathing process that took place as a result of maturation, water, and CO_2_ diffusions from the egg’s inner part to the surrounding atmosphere through the shell. It has been proposed that moisture loss increases due to the elongation of air cells in eggshells [27], which makes it easier for vapors to escape from the eggs.

Notable enlargement of air cells was observed during the 30 days of egg storage, indicating gradual aging in response to storage temperature and time. The mean value of air cell size of eggshell after 1 day of storage corresponded to 4.0 mm, impaling on their “extra freshness” according to the report of Grashorn [28]. After 10 days of storage, the increase made up 5%, corresponding to the cell size of 4.2 mm. The observed value did not exceed the critical value of 6.0 mm, indicating that the quality of eggs still complies with the regulations outlined by the EC regulation and belongs to grade A eggs [10]. However, after 25 and 28 days of egg storage, the size of the air cells was found to be exponentially enlarged by 25% and 82.5%, and the observed mean value corresponded to 5.0 and 7.3 mm, respectively. 

It has been anticipated that the increase in air cell size, moisture, and CO_2_ loss will weaken the structure and disrupt the integrity of eggshells. Consequently, the formation of empty spaces on the surface of eggshells will result in loss of strength. However, as seen in Table 1, no significant difference (*p* ≥ 0.05) in the eggshell breaking strength was observed neither after 10 nor 20 days of storage. However, a gradual reduction in eggshell breaking strength started to become apparent after 3 weeks of storage. As shown, up to 15.2% eggshell strength loss was observed after 21 days of storage. The reduction in strength after 30 days of storage was the most evident, which made an 18.1% loss of the initial strength. 

The albumen height is another factor to be critically evaluated during the quality screening of eggs since a strong correlation between albumen height and freshness of the eggs was repeatedly confirmed [29,30]. The pH changes from acidic to alkaline resulting from water and CO_2_ loss lead to albumen liquefaction and viscosity reduction [31]. Since albumen height strongly correlates with egg size, the Haugh unit (albumen height corrected for egg size) was also taken into consideration. A statistically significant (*p* ≤ 0.05) difference in albumen height was observed between the initial value and those after 10 days of storage. As seen, the decrease in albumin height after 10 days of egg storage was 18.5%; the values correspond to 4.9 and 4.0 mm for the 1st and 10th day of storage, respectively. The observed initial albumen height value is consistent with that reported by Silversides and Budgell for fresh eggs from Lohmann Brown laying hens [32]. A more substantial decrease in albumen height was observed during subsequent storage of eggs for an additional 20 days, corresponding to a reduction of 21.7% of the initial height. The percentage decrease is substantially lower than that reported by Lee et al. [33], observing a decrease of 32.8% in the initial albumen height. 

To better describe the freshness of the stored eggs, in this experiment, the Haugh unit was measured as proposed by Raymond Haugh in 1937 as one of the most important quality criteria, next to eggshell integrity and eggshell strength [24,34]. At the beginning of egg storage, the Haugh unit corresponded to 68.3, and the observed value is consistent with data reported in an earlier study by Grashorn [28]. However, similar to albumen height, the value of the Haugh units was significantly (*p* ≤ 0.05) influenced by storage duration, especially during the first two weeks of storage. As seen, on the 10th day of egg storage, the percentage decrease amounted to 11.4%. A further decrease was not observed, and passing 21 days of storage, percentage reduction corresponded to 11.0% loss compared with the initial value. Furthermore, for the next 9 days of storage, the Haugh unit remained almost unchanged. A fairly similar decrease in Haugh unit was reported by Hagan and Eichie [35], revealing a decrease of 10.4% after 20 days of storage for eggs from Lohmann Brown laying hens. 

As seen, no substantial depletion of egg albumen was revealed during the first 25 days of egg storage, and the quality corresponded to grade A according to the United States Department of Agriculture (USDA) [36]. However, on 28 days of storage, Haugh unit values were found to be lower than 60, moving the eggs to grade B due to weak and watery albumen. 

Overall, considering the results on physical–chemical attributes, the maximum shelf-life of the selected eggs could be defined as 28 days under room temperature (19 ± 1 °C) and RH of 50%, which is consistent with the EC regulations No 589/2008, which stipulate the “best before” date for shell eggs as 28 days after the laying date [10]. After this time, the albumen has undergone the most substantial thinning allowed to speculate the same scenario of quality deterioration in liquid whole egg products during storage in four types of packaging.

### 3.2. The Changes in Fatty Acid Profile of Liquid Whole Egg Products during 35 Days of Storage in Four Types of Packaging

**Figure 3:**
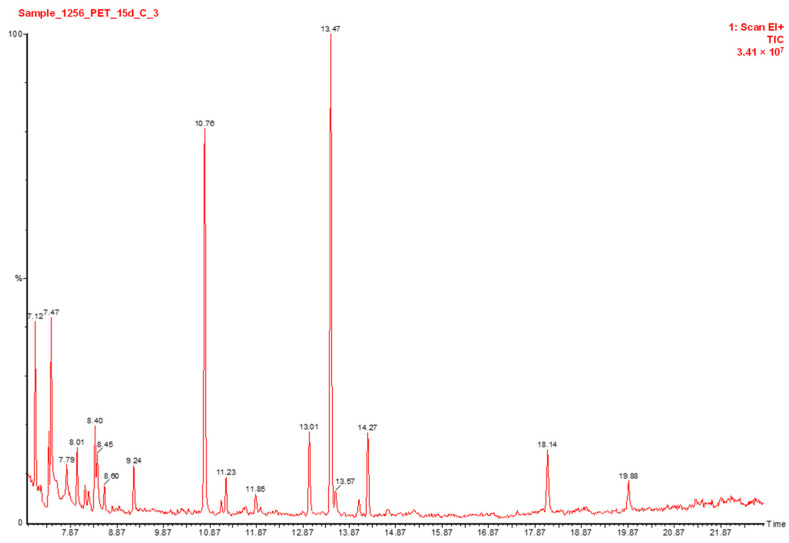
Chromatographic separation of major FAs recovered from the lipids of liquid whole egg product kept for 15 days in PET packaging: 10.76 min (C16:0—hexadecanoic acid), 11.23 min (C16:1n7c—hexadecenoic acid), 11.85 min (C17:0—heptadecanoic acid), 13.01 min (C18:0—octadecanoic acid), 13.47 min (C18:1n9c—octadecenoic acid), 13.57 min (C18:1n7t—11-octadecenoic acid), 14.27 min (C18:2n6c—octadecadienoic acid), 18.14 min (C22:0—docosanoic acid).

The FA profile of lipids recovered from LWEPs stored at 4 ± 1 °C using four types of packaging is depicted in Table A1 of Appendix A. In total, 28 FAs were identified and quantified, among which the prevalence of oleic acid (C18:1n9c) (42.45%), followed by palmitic acid (C16:0) (23.49%), linoleic acid (C18:2n6c) (8.86%), and stearic acid (C18:0) (6.64%) were observed at the initial stage of storage (Figure 3). The results are consistent with those of Zita et al. [37], indicating a fairly similar descending order of FA content recovered from organic eggs. As seen, storage resulted in a sharp decrease in FAs, and the percentage reduction was time and packaging type-dependent.

The loss of palmitic acid after 15 days of storage fluctuated in the range of 22.6–31.2%, with LWEPs stored in TR and DP having the highest, and LWEPs kept in PET packaging the lowest. The decrease in the content of palmitic acid is most likely due to the oxidation process caused by one of the disadvantages inherent to laminated materials, i.e., the ability of certain polymers to delaminate from the surface of paperboard and cause oxygen diffusion more extensively. The observed percentage decrease in the portion of palmitic acid was in line with that reported by Drabik et al. [38], indicating up to 17.3% palmitic acid loss in egg yolk kept in a paperboard box under room temperature (21 °C). However, the authors noted that there were no significant differences (*p* ≥ 0.05) between initial and final values for egg products kept in plastic boxes under room and refrigeration conditions (5 °C). Moreover, a fairly stable value of palmitic acid was reported by Hayat et al. [39], demonstrating no statistically significant difference (*p* ≥ 0.05) between the initial value and after 60 days of non-processed egg storage. However, the loss of palmitic acid after 35 days of storage was found to be in the range of 13.2–21.8%, which is much lower than values observed on the 15th day. The increase in the portion of palmitic acid after 35 days of storage can be associated presumably with the moisture loss caused by temperature and low relative air humidity in the storage chamber. However, another credible explanation has been given by Olafsson et al. [40], observing the interaction between FAs and packaging materials such as low-density polyethylene-foil-based composites that manifest by gradual sorption and desorption of FAs from packaging. The authors declared, however, that MUFAs were sorbed 2–4 times better than PUFAs. 

A similar trend of decrease was detected regarding the portion of stearic acid, the loss of which varied in the range of 8.0–16.7% and 9.2–16.0% after 15 and 35 days of storage, respectively. As shown, the highest reduction of stearic acid after 15 days of storage was observed in LWEP stored in DP (16.7%), followed by TR (13.3%), and HDPE (12.3%). PET packaging demonstrated a relatively better preservation ability, corresponding to 8.0% of stearic acid loss only. However, an additional 20 days of storage revealed continuous degradation of FAs. As seen, the most substantial reduction was in LWEP kept in PET (16.0%) and HDPE (11.3%), indicating a gradual oxidation process. In turn, the percentage increase in stearic acid content by 17.0% and 9.0% compared with the values of 15 days of storage was observed for LWEPs maintained in TR and DP packaging, respectively. The adverse effect of PET and HDPE has been demonstrated by Zarazir et al. [41], revealing both accumulations of lipid peroxidation products and intensive sorption of FAs during storage of olive oil caused by a non-uniform surface and the presence of free volumes within amorphous regions of the polymers. However, an increase in the content of stearic acid in LWEPs maintained in TR and DP could also be a matter of moisture loss and LWEP shrinkage, contributing to the concentration of soluble solids content. The results of this study revealed relatively higher water permeability of multi-layer packaging, i.e., TR and DP, which contain low-density polyethylene (LDPE) and EVOH polymer layers, respectively. This observation is consistent with data provided by Bastarrachea et al. [42], revealing relatively poor water vapor barrier properties of such plastic films as LDPE and EVOH compared to HDPE and PET. Meanwhile, the presence of hydroxyl groups in the vinyl alcohol unit of EVOH makes this material highly hydrophilic and thus sensitive to water [43].

The portion of oleic acid in LWEP stored for 15 days decreased significantly (*p* ≤ 0.05), corresponding to a percentage loss of 23.2–30.2%, with LWEP stored in HDPE packaging having the lowest loss and LWEPs kept in TR and DP the highest. The results are not consistent with data reported by Drabik et al. [38], demonstrating a decrease in the portion of oleic acid only by 14.9% in egg yolk kept in paperboard packaging under room temperature for 28 days, and an increase of 8.8% in product kept under refrigeration conditions. After 35 days of storage, the portion of oleic acid decreased by 18.8–25.7% compared with the initial value. The most substantial reduction was observed in LWEP stored in PET packaging (25.7%), followed by TR (23.4%). As in the case of stearic acid, the concentration of oleic acid in LWEPs kept in TR and DP packaging increased by 9.7% and 12.3% compared with the date after 15 days of storage. The percentage loss amounted to 23.4% and 16.8%, respectively. A fairly similar reduction in the content of oleic acid was observed by Zarazir et al. [41], revealing percentage loss of this acid by 32.7% and 29.6% for extra virgin olive oil stored in PET and HDPE packaging under ambient temperature for 3 months. Substantially better maintenance of oleic acid was achieved by using glass (6.6%) and bio-based polylactic acid (PLA) packaging (18.2%).

The content of MUFA linoleic acid was found to be dependent on storage time and type of storage packaging. After 15 days of LWEP storage, the highest loss was observed in TR and DP products, the percentage loss corresponding to 30.5% and 31.2%, respectively. After 35 days of storage, the portion of linoleic acid decreased by 22.7–27.9% compared with the initial value. The lowest loss was observed for LWEP kept in DP (22.7%), while the highest was in PET (27.9%) and TR (27.7%). However, there were no significant differences (*p* ≥ 0.05) in the content of linoleic acid between 15 and 35 days of LWEPs storage for PET packaging. Unfortunately, no direct comparison of the observed values could be performed due to existing limitations in the literature. However, Drabik et al. [38] pointed to an increase in the content of linoleic acid by 16.7% and a decrease of 16.1% in egg yolk stored in paperboard under room and refrigerated storage conditions for 28 days, respectively. The authors reported exactly the opposite values regarding plastic packaging, where the reduction in the content of linoleic acid in egg yolk under room and refrigerated conditions amounted to 16.7% and only 2.9%, respectively.

Overall, the content of FAs in LWEPs was affected by both storage time and packaging material. Without reference to the increase in the content of individual FAs that is likely explained by the moisture loss and shrinkage of the stored products, the better preservation of MUFAs was achieved by PET, followed by HDPE packaging. Meanwhile, a statistically significant advantage of PET over other packaging materials has been confirmed regarding the maintenance of PUFAs during 35 days of LWEPs storage.

### 3.3. The Changes in Amino Acid Profile of Liquid Whole Egg Products during 35 Days of Storage in Four Types of Packaging

In human nutrition, AAs play an important role, as they are involved in many vital processes such as the synthesis of proteins, hormones, and neurotransmitters [44]. The availability of essential AAs in whole eggs makes this product unique from a nutritional standpoint. 

The analysis of AAs at the initial stage of LWEPs storage revealed the presence of 17 AA representatives, the values of which fluctuated in the range from 0.21 to 1.15 g 100 g^−1^ (Table A2 of Appendix B). The prevalence of glutamic, followed by aspartic acids, leucine, and lysine was highlighted, the values corresponding to 1.15, 0.92, 0.75, and 0.68 g 100 g^−1^, respectively. The observed values are consistent with those reported by Attia et al. [45], also indicating the prevalence of glutamic acid over other AAs in whole eggs originating from Single Comb White Leghorn laying hens. 

After 15 days of storage, the concentrations of individual AAs were found in the range from 0.21 to 1.33 g 100 g^−1^, contributing to the total amount of AAs from 8.79 to 9.68 g 100 g^−1^. As seen, an increase in the total content of AAs was observed for all packaging materials, except for PET. The most substantial increase was found in the LWEPs kept in DP (an average increase of 12.7%), followed by HDPE (9.62% increase), and TR (8.3% increase). Fairly stable AA values were observed in LWEP stored in PET packaging (an average decrease of 0.4%). The most obvious increase in individual AAs was noted for cysteine, histidine, and lysine, making an average percentage increase for PET, TR, HDPE, and DP of 0.0%, 13.0%, and 3.4%, and 20.3%, respectively. 

After 35 days of storage, the concentration of individual AAs similar to the 15th day of storage fluctuated in the range from 0.21 to 1.32 g 100 g^−1^. A similar ascending trend of AAs increase was highlighted compared with the initial AA values. The most substantial increase was observed for individual AAs such as histidine, cysteine, and arginine, corresponding to the percentage increase of 20.6%, 14.3%, and 14.2%, respectively. Among non-essential AAs, alanine was found to be the most stable since only a 4.0% difference (average for all packaging materials) between the initial and 35th day of storage was observed. However, methionine and leucine, as representatives of essential AAs, demonstrated comparable stability over 35 days of storage, corresponding only to 0.8% and 0.4% differences (average for all packaging materials) between initial values and the 35th day of storage. On average, the increase in the total AA content for HDPE, TR, and DP corresponded to 9.0%, 9.9%, and 9.2%. The moisture loss caused by exfoliation of the internal polymer layers presented in TR and the hydrophilic nature of the EVOH layer in DP were perhaps the main causes for such an increase, as also highlighted for FA values. A similar observation has been made by Shin and Selke [46], highlighting that EVOH is a readily water-soluble polymer and loses its gas barrier properties under humid conditions, which greatly limits its applications within food packaging. In turn, due to better barrier properties over other packaging materials, PET demonstrated better preservation of AAs since only a 2.1% decrease was observed during 35 days of storage.

### 3.4. Total Plate Count Growth Dynamics during Whole Egg Product Storage of 35 Days in Four Types of Packaging

**Figure 4:**
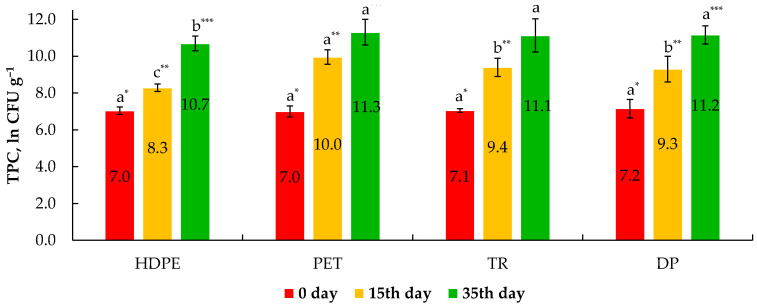
Total plate count growth dynamics during liquid whole egg product storage for 35 days in four types of packaging. Note: Values are means ± SD of triplicates (*n* = 3). Means within the same storage period (*—0 day, **—15 days, ***—35 days) with different superscript letters (^a, b, c^) are significantly different at *p* ≤ 0.05. HDPE21—high-density polyethylene; PET21—polyethylene terephthalate; TR21—Tetra Rex bio-based; DP21—Doypack.

Apart from physical–chemical attributes and nutritional aspects, microbiological quality also determines the safety and suitability of food materials for consumption [47]. The presence of microorganisms in the initial steps of egg production due to inadequate sanitary conditions risks further microorganism development in the retail environment both externally and internally. The role of total plate count (TPC) is of significant interest because it is linked with egg safety and product shelf-life [48]. The dynamic of TPC development in LWEPs during storage using four types of packaging is depicted in Figure 4. As seen, the average number of TPC at the initial stage of storage fluctuated in the range from 7.0 to 7.2 ln CFU g^−1^.

The observed values comply with the criteria outlined by the EC regulations No 589/2008 and No 2073/2005, indicating a maximum allowed limit of 10^5^ CFU g^−1^ or mL^−1^ [10,11]. Pasteurization of LWEPs at 68 ± 1 °C for 6 min ensured complete inhibition of Coliform bacteria since no presence of any of its representatives was found at the initial stage of storage. On the 15th day of LWEPs storage at 4 ± 1 °C, there was a notable increase in the number of viable count microorganisms, the range of which fluctuated within 8.3 to 10.0 ln CFU g^−1^. The observed TPC values are consistent with those of Miller et al. [49], indicating the effectiveness of LWEPs pasteurization at 68 °C for 2.5 min. On the 35th day of storage, the values for the content of the TPC increased by 3.7 to 4.3 ln CFU g^−1^, with LWEP maintained in HDPE packaging having the lowest increase and LWEP kept in PET the highest. Despite such a statistically significant (*p* ≤ 0.05) increase in the content of TCP, stored LWEPs still met microbiological quality criteria and are considered to be safe for consumption.

## 4. Conclusions

The initial screening of the physical attributes of hen eggs collected for the preparation of LWEPs revealed conformity of physical attributes after 25 days of storage. The quantitative and qualitative analyses of LWEPs by GC-MS and HPLC systems indicated that the content of FAs was affected both by storage time and type of packaging. After 15 days of storage, the most significant decrease was revealed in the content of linoleic and oleic acids in LWEPs stored in DP and TR, corresponding to percentage losses of 31.2% and 30.2% of the initial values, respectively. However, during the next 20 days of storage, an increase in FA content was observed in LWEPs kept in TR and DP, while less pronounced changes were found in PET and HDPE packaging. Similar to FAs, the most substantial fluctuations in the content of AAs appeared during 15 days of LWEPs storage, while their content was less affected during the additional 20 days of storage. Due to better barrier properties, PET packaging demonstrated better preservation of AAs since only a 2.1% decrease was observed during 35 days of storage. Relative fluctuations in the content of both FAs and AAs in LWEPs stored in TR and DP packaging were most likely associated with exfoliation of composite layers of packaging, which resulted in extensive moisture loss and LWEP shrinkage. From a microbiological standpoint, after 35 days of storage, all LWEPs met microbiological quality criteria of not exceeding a maximum allowed limit of 10^5^ CFU g^−1^.

## Figures and Tables

**Table 1:** Changes in egg physical attributes during storage period of 30 days.

Attribute	Days of Storage										
	1	4	7	10	14	16	18	21	25	28	30
Weight, g	57.7 ± 1.7 ^a^	56.2 ± 1.7 ^a^	55.6 ± 1.7 ^b^	55.2 ± 1.6 ^b^	53.8 ± 1.6 ^c^	54.3 ± 1.6 ^b^	54.4 ± 1.6 ^b^	53.4 ± 1.6 ^c^	54.6 ± 1.6 ^b^	54.3 ± 1.6 ^b^	51.8 ± 1.5 ^c^
Air cell size, mm	4.0 ± 0.1 ^c^	4.2 ± 0.13 ^c^	4.1 ± 0.1 ^c^	4.2 ± 0.1 ^c^	4.5 ± 0.13 ^c^	4.7 ± 0.1 ^b^	4.4 ± 0.13 ^c^	4.9 ± 0.1 ^b^	5.0 ± 0.1 ^b^	7.3 ± 0.2 ^a^	7.5 ± 0.2 ^a^
Eggshell strength, kg cm^3^	5.3 ± 0.1 ^a^	5.3 ± 0.1 ^a^	4.9 ± 0.1 ^b^	5.9 ± 0.2 ^a^	5.4 ± 0.2 ^a^	5.4 ± 0.1 ^a^	5.1 ± 0.1 ^a^	4.5 ± 0.1 ^c^	4.4 ± 0.1 ^c^	4.4 ± 0.1 ^c^	4.3 ± 0.1 ^c^
Albumen height, mm	4.9 ± 0.1 ^a^	4.7 ± 0.1 ^a^	4.3 ± 0.1 ^a^	4.0 ± 0.1 ^c^	4.2 ± 0.1 ^b^	4.2 ± 0.1 ^b^	4.2 ± 0.1 ^b^	3.9 ± 0.1 ^c^	3.9 ± 0.1 ^c^	3.8 ±0.1 ^c^	3.6 ± 0.1 ^c^
Haugh units	68.3 ± 2.0 ^a^	67.1 ± 2.0 ^a^	63.4 ± 1.9 ^b^	60.5 ± 1.8 ^c^	63.4 ± 1.9 ^b^	63.4 ± 1.9 ^b^	63.2 ± 1.9 ^b^	60.8 ± 1.8 ^c^	60.7 ± 1.8 ^c^	58.0 ±1.7 ^c^	57.0 ± 1.7 ^c^
Dry matter, %	24.0 ± 0.7 ^b^	24.5 ± 0.7 ^b^	24.9 ± 0.7 ^b^	24.3 ± 0.7 ^b^	24.6 ± 0.7 ^b^	24.2 ± 0.7 ^b^	24.9 ± 0.7 ^b^	24.8 ± 0.7 ^b^	25.2 ± 0.8 ^a^	25.3 ±0.8 ^a^	25.2 ± 0.7 ^a^
pH	7.8 ± 0.2 ^b^	7.9 ± 0.2 ^a^	7.9 ± 0.2 ^a^	7.8 ± 0.2 ^b^	7.8 ± 0.2 ^b^	7.9 ± 0.2 ^a^	7.9 ± 0.2 ^a^	7.9 ± 0.2 ^a^	7.8 ± 0.2 ^b^	7.9 ±0.2 ^a^	7.9 ± 0.2 ^a^

Note: Values are means ± SD of twenty replicates (*n* = 20). Note. Means within the same row with different superscript letters (^a, b, c^) are significantly different at *p* ≤ 0.05; DW—dry weight; SD—standard deviation.

## Data Availability

The data sets and analysis of the study are available from the corresponding author upon reasonable request.

## Appendix A

**Table A1:** Fatty acid profile of liquid whole egg lipids in relation to the time of storage and type of packaging, % FW.

Fatty Acid	Abbreviation	Initial	HDPE	HDPE	PET	PET	TR	TR	DP	DP
		(0 Day)	(15th Day)	(35th Day)	(15th Day)	(35th Day)	(15th Day)	(35th Day)	(15th Day)	(35th Day)
Tridecanoic acid	C13:0	2.2 ± 0.1	6.3 ± 0.3	6.7 ± 0.3	5.6 ± 0.3	7.3 ± 0.4	5.3 ± 0.3	6.8 ± 0.3	6.3 ± 0.3	6.7 ± 0.3
Tetradecanoic acid	C14:0	n.d.	n.d.	n.d.	n.d.	n.d.	n.d.	n.d.	n.d.	n.d.
Tetradecenoic acid	C14:1	n.d.	n.d.	n.d.	n.d.	n.d.	n.d.	n.d.	n.d.	n.d.
Pentadecanoic acid	C15:0	1.9 ± 0.0	n.d.	5.6 ± 0.3	n.d.	n.d.	n.d.	n.d.	n.d.	6.0 ± 0.3
Pentadecanoic acid	C15:1	BLQ	5.3 ± 0.3	5.2 ± 0.3	5.0 ± 0.2	5.6 ± 0.3	4.4 ± 0.2	BLQ	5.1 ± 0.2	0.0
Hexadecanoic acid	C16:0	23.5 ± 1.1	17.5 ± 0.9	18.9 ± 0.9	18.2 ± 0.9	18.7 ± 0.9	16.6 ± 0.8	20.4 ± 1.0	16.2 ± 0.8	18.4 ± 0.9
Hexadecenoic acid	C16:1n7c	0.7 ± 0.0	BLQ	BLQ	BLQ	BLQ	BLQ	BLQ	BLQ	BLQ
Heptadecanoic acid	C17:0	0.8 ± 0.0	2.7 ± 0.1	2.4 ± 0.1	2.4 ± 0.1	3.3 ± 0.2	2.3 ± 0.1	2.6 ± 0.1	2.5 ± 0.1	2.3 ± 0.1
Heptadecenoic acid	C17:1	BLQ	1.4 ± 0.1	BLQ	1.1 ± 0.0	BLQ	1.1 ± 0.0	BLQ	BLQ	BLQ
Octadecanoic acid	C18:0	6.6 ± 0.3	5.8 ± 0.3	5.9 ± 0.3	6.1 ± 0.3	5.6 ± 0.3	5.8 ± 0.3	6.7 ± 0.3	5.5 ± 0.3	6.0 ± 0.3
Octadecenoic acid	C18:1n9t	n.d.	n.d.	n.d.	n.d.	n.d.	n.d.	n.d.	n.d.	n.d.
Octadecenoic acid	C18:1n9c	42.5 ± 2.1	32.6 ± 1.6	33.2 ± 1.6	32.8 ± 1.6	31.5 ± 1.6	29.7 ± 1.5	32.5 ± 1.6	31.5 ± 1.6	35.3 ± 1.8
Octadecenoic acid	C18:1n7t	0.5 ± 0.0	BLQ	0.2 ± 0.0	1.2 ± 0.1	BLQ	0.3 ± 0.0	0.3 ± 0.0	0.1 ± 0.0	BLQ
Octadecadienoic acid	C18:2n6t	n.d.	n.d.	n.d.	n.d.	n.d.	n.d.	n.d.	n.d.	n.d.
Octadecadienoic acid	C18:2n6c	8.9 ± 0.4	6.5 ± 0.3	6.7 ± 0.3	6.4 ± 0.3	6.4 ± 0.3	6.2 ± 0.3	6.4 ± 0.3	6.1 ± 0.3	6.9 ± 0.3
Octadecatrienoic acid	C18:3n6	n.d.	n.d.	n.d.	n.d.	n.d.	n.d.	n.d.	n.d.	n.d.
Octadecatrienoic acid	C18:3n3	0.6 ± 0.0	1.3 ± 0.1	n.d.	1.5 ± 0.1	1.6 ± 0.1	1.7 ± 0.1	1.2 ± 0.0	1.6 ± 0.1	1.7 ± 0.1
Eicosanoic acid	C20:0	n.d.	n.d.	n.d.	n.d.	n.d.	0.2 ± 0.0	n.d.	n.d.	n.d.
CLA, Octadecadienoic acid	C18:2	0.4 ± 0.0	BLQ	BLQ	BLQ	BLQ	BLQ	BLQ	BLQ	BLQ
CLA, Octadecadienoic acid	C18:2	0.5 ± 0.0	0.9 ± 0.0	n.d.	0.9 ± 0.0	n.d.	1.1 ± 0.0	n.d.	1.4 ± 0.1	n.d.
Eicosenoic acid	C20:1n9c	0.3 ± 0.0	0.5 ± 0.0	BLQ	1.7 ± 0.1	0.9 ± 0.0	0.7 ± 0.0	0.4 ± 0.0	0.5 ± 0.0	1.8 ± 0.1
Heneicosanoic acid	C21:0	BLQ	1.3 ± 0.1	BLQ	BLQ	BLQ	1.3 ± 0.1	BLQ	1.5 ± 0.1	1.3 ± 0.1
Eicosadienoic acid	C20:2n6c	n.d.	n.d.	n.d.	n.d.	n.d.	1.0 ± 0.0	n.d.	1.8 ± 0.1	n.d.
Eicosatrienoic acid	C20:3n6c	0.4 ± 0.0	0.6 ± 0.0	0.5 ± 0.0	0.5 ± 0.0	1.2 ± 0.1	BLQ	BLQ	0.7 ± 0.0	BLQ
Eicosatetraenoic acid	C20:4n6c	1.2 ± 0.0	0.3 ± 0.0	0.6 ± 0.0	0.4 ± 0.0	BLQ	2.6 ± 0.1	1.7 ± 0.1	0.3 ± 0.0	BLQ
Eicosatrienoic acid	C20:3n3c	0.3 ± 0.0	BLQ	0.8 ± 0.0	0.8 ± 0.0	BLQ	BLQ	0.7 ± 0.0	0.7 ± 0.0	0.8 ± 0.0
Docosanoic acid	C22:0	2.5 ± 0.1	11.1 ± 0.5	11.1 ± 0.5	10.5 ± 0.5	12.4 ± 0.6	11.0 ± 0.5	10.3 ± 0.5	10.1 ± 0.5	12.9 ± 0.6
Docosenoic acid	C22:1n9c	0.6 ± 0.0	0.4 ± 0.0	BLQ	0.2 ± 0.0	BLQ	0.1 ± 0.0	BLQ	0.3 ± 0.0	BLQ
Eicosapentaenoic acid	C20:5n3c	0.2 ± 0.0	1.2 ± 0.0	BLQ	0.5 ± 0.0	BLQ	1.7 ± 0.1	0.8 ± 0.0	1.3 ± 0.1	BLQ
Tricosanoic acid	C23:0	0.6 ± 0.0	BLQ	1.9 ± 0.1	BLQ	3.0 ± 0.1	1.9 ± 0.1	2.3 ± 0.1	2.5 ± 0.1	BLQ
Docosadienoic acid	C22:2n6	n.d.	0.3 ± 0.0	0.2 ± 0.0	0.7 ± 0.0	0.8 ± 0.0	0.1 ± 0.0	1.7 ± 0.1	0.7 ± 0.0	n.d.
Tetracosanoic acid	C24:0	0.6 ± 0.0	1.2 ± 0.0	n.d.	0.5 ± 0.0	1.8 ± 0.1	1.4 ± 0.1	1.8 ± 0.1	0.6 ± 0.0	n.d.
Tetracosenoic acid	C24:1n9c	0.2 ± 0.0	0.1 ± 0.0	BLQ	0.5 ± 0.0	BLQ	1.4 ± 0.1	0.3 ± 0.0	BLQ	BLQ
Docosahexaenoic acid	C22:6n3c	1.2 ± 0.1	3.0 ± 0.1	BLQ	2.8 ± 0.1	BLQ	2.5 ± 0.1	3.0 ± 0.1	2.7 ± 0.1	BLQ
**∑_SFAs_**		41.3 ± 2.1	45.9 ± 2.3	52.5 ± 2.6	43.3 ± 2.1	52.0 ± 2.6	45.7 ± 2.3	51.0 ± 2.5	45.3 ± 2.3	53.6 ± 2.7
**∑_MUFAs_**		44.9 ± 2.2	40.2 ± 2.0	38.7 ± 1.9	42.3 ± 2.1	38.0 ± 1.9	37.5 ± 1.9	33.5 ± 1.7	37.5 ± 1.9	37.1 ± 1.8
**∑_PUFAs_**		13.8 ± 0.7	13.9 ± 0.7	8.8 ± 0.4	14.4 ± 0.7	9.9 ± 0.5	16.8 ± 0.8	15.5 ± 0.8	17.3 ± 0.9	9.3 ± 0.5

Note: Values are means ± SD of triplicates (*n* = 3). SFAs—saturated fatty acids; MUFAs—monounsaturated fatty acids; PUFAs—polyunsaturated fatty acids; CLA—conjugated linoleic acid; HDPE—high-density polyethylene; PET—polyethylene terephthalate TR—Tetra Rex^®^ Bio-based; DP—Doypak with ethylene vinyl alcohol layer; BLQ—below limit of quantification; SD—standard deviation; n.d.—not detected.

## Appendix B

**Table A2:** Amino acid profile of whole eggs in relation to the time of storage and type of packaging, g 100 g^−1^ FW.

Amino Acid	Initial	HDPE	HDPE	PET	PET	TR	TR	DP	DP
	(0 Day)	(15th Day)	(35th Day)	(15th Day)	(35th Day)	(15th Day)	(35th Day)	(15th Day)	(35th Day)
Alanine	0.50 ± 0.02 ^b^	0.52 ± 0.03 ^a^	0.53 ± 0.01 ^a^	0.49 ± 0.01 ^b^	0.49 ± 0.01 ^b^	0.52 ± 0.06 ^a^	0.53 ± 0.04 ^a^	0.53 ± 0.06 ^a^	0.53 ± 0.04 ^a^
Arginine	0.54 ± 0.08 ^c^	0.59 ± 0.05 ^b^	0.60 ± 0.04 ^b^	0.54 ± 0.02 ^c^	0.53 ± 0.02 ^c^	0.60 ± 0.05 ^b^	0.62 ± 0.05 ^a^	0.58 ± 0.06 ^b^	0.63 ± 0.05 ^a^
Aspartic acid	0.92 ± 0.04 ^c^	1.01 ± 0.08 ^a^	0.98 ± 0.09 ^b^	0.91 ± 0.05 ^c^	0.88 ± 0.07 ^d^	0.99 ± 0.09 ^a,b^	0.99 ± 0.09 ^a,b^	1.00 ± 0.13 ^a^	1.01 ± 0.12 ^a^
Cystine	0.21 ± 0.01 ^d^	0.24 ± 0.01 ^b,c^	0.23 ± 0.01 ^c^	0.21 ± 0.03 ^d^	0.21 ± 0.02 ^d^	0.24 ± 0.03 ^b,c^	0.23 ± 0.03 ^c^	0.25 ± 0.03 ^a,b^	0.26 ± 0.03 ^a^
Phenylalanine	0.48 ± 0.03 ^c^	0.52 ± 0.01 ^a,b^	0.51 ± 0.02 ^b^	0.48 ± 0.06 ^c^	0.49 ± 0.05 ^c^	0.52 ± 0.04 ^a,b^	0.53 ± 0.04 ^a^	0.52 ± 0.04 ^a^	0.53 ± 0.04 ^a^
Glycine	0.30 ± 0.05 ^b^	0.32 ± 0.03 ^a^	0.32 ± 0.02 ^a^	0.29 ± 0.06 ^b^	0.29 ± 0.02 ^b^	0.32 ± 0.04 ^a^	0.32 ± 0.04 ^a^	0.32 ± 0.04 ^a^	0.33 ± 0.04 ^a^
Glutamic acid	1.15 ± 0.03 ^e^	1.30 ± 0.09 ^b^	1.28 ± 0.11 ^c^	1.15 ± 0.13 ^e^	1.14 ± 0.12 ^e^	1.26 ± 0.16 ^d^	1.32 ± 0.11 ^a^	1.28 ± 0.11 ^c^	1.33 ± 0.19 ^a^
Histidine	0.21 ± 0.05 ^c^	0.24 ± 0.01 ^b^	0.25 ± 0.02 ^a^	0.21 ± 0.02 ^c^	0.21 ± 0.03 ^c^	0.24 ± 0.03 ^b^	0.25 ± 0.03 ^a,b^	0.24 ± 0.03 ^b^	0.26 ± 0.03 ^a^
Isoleucine	0.45 ± 0.02 ^c^	0.48 ± 0.01 ^b^	0.49 ± 0.04 ^a,b^	0.45 ± 0.07 ^d^	0.43 ± 0.05 ^d^	0.48 ± 0.04 ^b^	0.50 ± 0.06 ^a^	0.49 ± 0.05 ^a,b^	0.50 ± 0.04 ^a^
Leucine	0.75 ± 0.08 ^c^	0.81 ± 0.06 ^a,b^	0.81 ± 0.07 ^a,b^	0.74 ± 0.08 ^d^	0.71 ± 0.08 ^e^	0.80 ± 0.07 ^b,c^	0.79 ± 0.08 ^c^	0.81 ± 0.07 ^a,b^	0.82 ± 0.07 ^a^
Lysine	0.68 ± 0.05 ^c^	0.76 ± 0.09 ^a,b^	0.73 ± 0.07 ^c^	0.68 ± 0.04 ^d^	0.67 ± 0.07 ^d^	0.75 ± 0.08 ^b^	0.74 ± 0.08 ^b,c^	0.74 ± 0.06 ^b,c^	0.77 ± 0.06 ^a^
Methionine	0.31 ± 0.01 ^a,b^	0.33 ± 0.01 ^a,b^	0.32 ± 0.05 ^b^	0.31 ± 0.06 ^b,c^	0.30 ± 0.02 ^c^	0.32 ± 0.03 ^b^	0.31 ± 0.04 ^b,c^	0.32 ± 0.04 ^b^	0.34 ± 0.04 ^a^
Proline	0.36 ± 0.01 ^c^	0.39 ± 0.02 ^a^	0.40 ± 0.03 ^a^	0.36 ± 0.02 ^c^	0.36 ± 0.05 ^c^	0.37 ± 0.04 ^b,c^	0.41 ± 0.05 ^a^	0.38 ± 0.05 ^b^	0.41 ± 0.05 ^a^
Serine	0.64 ± 0.02 ^d^	0.71 ± 0.05 ^a,b^	0.70 ± 0.04 ^b,c^	0.64 ± 0.05 ^e^	0.60 ± 0.05 ^e^	0.70 ± 0.06 ^b,c^	0.69 ± 0.08 ^c,d^	0.68 ± 0.06 ^d^	0.72 ± 0.06 ^a^
Tyrosine	0.37 ± 0.01 ^c^	0.41 ± 0.05 ^b^	0.44 ± 0.03 ^a^	0.37 ± 0.01 ^c^	0.36 ± 0.03 ^c^	0.41 ± 0.03 ^b^	0.44 ± 0.05 ^a^	0.44 ± 0.03 ^a^	0.43 ± 0.05 ^a^
Threonine	0.40 ± 0.02 ^c^	0.44 ± 0.03 ^b^	0.46 ± 0.07 ^a^	0.40 ± 0.02 ^c^	0.40 ± 0.05 ^c^	0.43 ± 0.03 ^b^	0.46 ± 0.05 ^a^	0.45 ± 0.04 ^a^	0.44 ± 0.05 ^b^
Valine	0.56 ± 0.05 ^c^	0.61 ± 0.09 ^b^	0.58 ± 0.03 ^c^	0.56 ± 0.05 ^d^	0.57 ± 0.06 ^c,d^	0.61 ± 0.07 ^a^	0.58 ± 0.06 ^c^	0.62 ± 0.05 ^a,b^	0.63 ± 0.06 ^a^
**EAAs**	3.84 ± 0.31 ^d^	4.19 ± 0.31 ^b^	4.15 ± 0.37 ^c^	3.83 ± 0.40 ^d^	3.78 ± 0.41 ^e^	4.15 ± 0.39 ^c^	4.16 ± 0.44 ^c^	4.29 ± 0.38 ^a^	4.19 ± 0.39 ^b^
**NEAAs**	4.99 ± 0.27 ^d^	5.49 ± 0.41 ^c^	5.48 ± 0.38 ^c^	4.96 ± 0.38 ^d^	4.86 ± 0.39 ^e^	5.41 ± 0.56 ^d^	5.55 ± 0.54 ^b^	5.65 ± 0.57 ^a^	5.46 ± 0.63 ^b^
**EAAs/** **NEAAs**	0.77 ^a^	0.76 ^a^	0.76 ^a^	0.77 ^a^	0.78 ^a^	0.77 ^a^	0.75 ^a^	0.76 ^a^	0.77 ^a^
**Total**	8.83 ± 0.58 ^e^	9.68 ± 0.72 ^c^	9.63 ± 0.75 ^c^	8.79 ± 0.78 ^f^	8.64 ± 0.80 ^g^	9.56 ± 0.95 ^d^	9.71 ± 0.98 ^b^	9.94 ± 0.95 ^a^	9.65 ± 1.02 ^c^

Note: Values are means ± SD of triplicates (*n* = 3). Means within the same row with different superscript letters (^a, b, c, d, e, f, g^) are significantly different at *p* ≤ 0.05. HDPE—high-density polyethylene; PET—polyethylene terephthalate TR—Tetra Rex^®^ Bio-based; DP—Doypak with ethylene vinyl alcohol layer; EAAs—total essential amino acids; NEAAs—total non-essential amino acids; EAAs/NEAAs—the ratio of total essential to non-essential amino acids; FW—fresh weight; SD—standard deviation; n.d.—not detected.
